# FMR1 KH0-KH1 domains coordinate m^6^A binding and phase separation in Fragile X syndrome

**DOI:** 10.1016/j.yexcr.2025.114664

**Published:** 2025-06-28

**Authors:** Xian Zhou, Chen-Jun Guo, Rui Wang, Yi-Lan Li, Tianyi Zhang, Zhuangyi Qiu, Shaorong Gao, Ji-Long Liu, Yawei Gao

**Affiliations:** a State Key Laboratory of Cardiology and Medical Innovation Center, Department of Reproductive Medicine Center, Shanghai East Hospital, Frontier Science Center for Stem Cell Research, School of Life Sciences and Technology, Tongji University, Shanghai, 200092, China; b School of Life Science and Technology, ShanghaiTech University, Shanghai, China; c Clinical and Translational Research Center of Shanghai First Maternity and Infant Hospital, Frontier Science Center for Stem Cell Research, School of Life Sciences and Technology, Tongji University, Shanghai, China; d Department of Physiology, Anatomy and Genetics, University of Oxford, Oxford, OX1 3PT, United Kingdom; e Sycamore Research Institute of Life Sciences, Shanghai, 201203, China

**Keywords:** FMR1, KH0 domain, m^6^A RNA, Phase separation, Fragile X syndrome

## Abstract

Fragile X messenger ribonucleoprotein 1 (FMR1) regulates neurodevelopment through m^6^A RNA interactions, yet the domain-specific roles of KH0 and KH1 in RNA binding and disease pathogenesis remain poorly understood. Using mutagenesis and AlphaFold3 structural modeling, we identify KH1 as the primary m^6^A-binding interface, while the KH0 domain (particularly Arg138) modulates liquid-liquid phase separation (LLPS). Pathogenic mutations in KH0 impair RNA binding and promote aberrant LLPS aggregation, whereas m^6^A-modified RNA suppresses LLPS formation at KH0. Structural simulations uncover synergistic interactions between KH0 and KH1 mediated by hydrophobic and electrostatic networks. These domain-specific cooperations establish a mechanistic link between m^6^A dysregulation, pathological phase separation, and Fragile X syndrome pathogenesis. Our findings nominate KH0 as a potential therapeutic target for RNA-driven neurodevelopmental disorders.

## 1 Introduction

Fragile X syndrome (FXS), the most common inherited form of intellectual disability, is caused by mutations or epigenetic changes that disrupt fragile X messenger ribonucleoprotein 1 (FMR1) [1,2], an X-linked gene essential for normal neurodevelopment. Loss of FMR1 function leads to characteristic neurological and cognitive deficits, with additional contributions from impaired FMR1-RNA interactions [3,4].

The FMR1 protein contains multiple functional domains including an N-terminal region, multiple KH RNA-binding domains, and a C-terminal RGG box [5,6]. A recently identified KH0 domain located upstream of KH1 has been implicated in FXS pathogenesis, with patient mutations in this region disrupting protein function [7,8]. While the RGG box mediates specific mRNA binding, the KH domains play a more complex role in regulating RNA interactions and protein function [9,10]. Notably, mutations in the KH2 domain, particularly at position I304, have been directly linked to intellectual disability, demonstrating the functional importance of these domains [11,12]. The newly characterized KH0 domain (residues 126–202) contains critical residues like Arg138 that when mutated contribute to both intellectual disability and epilepsy, highlighting its pathophysiological significance [8].

Beyond its structural domains, FMR1’s molecular function is deeply connected to RNA modifications. N6-methyladenosine (m^6^A), the most prevalent mRNA modification, regulates key aspects of RNA metabolism including stability, splicing, and translation [13–15]. This modification is recognized by specific reader proteins that mediate its cellular effects [16,17]. The YTH domain family, particularly YTHDF2, serves as a well-characterized m^6^A reader that directs modified transcripts for degradation through interactions with processing bodies [18–20].

Recent studies have established FMR1 as an m^6^A reader protein, revealing a direct connection between mRNA modifications and neurodevelopmental disorders including autism spectrum disorder (ASD). Functional studies in model organisms demonstrate that FMR1-m^6^A interactions regulate mRNA stability and translation, influencing neural development [21,22].

In this study, we combined biochemical assays with structural analyses to investigate how FMR1 domains mediate RNA binding and phase separation. Using electrophoretic mobility shift assays (EMSA) and surface plasmon resonance (SPR), we characterized mutant proteins with altered RNA-binding properties. Microscopy and electron microscopy approaches allowed us to directly observe changes in protein phase behavior. Our results identify critical residues within the KH0 domain that regulate both RNA binding and phase separation, providing new insights into FXS and ASD mechanisms. Furthermore, our integrative approach combining experimental data with computational modelingsuggests that m^6^A modification can alter RNA structure in ways that modulate protein binding, offering a potential regulatory mechanism for gene expression.

This work advances our understanding of how FMR1 integrates multiple layers of RNA regulation through its domain architecture, with important implications for neurodevelopmental disorders. The identification of KH0 as a key functional domain provides a potential target for therapeutic intervention in FXS and related conditions.

## 2 Results

### 2.1 Interaction between FMR1 and RNA

Recent advances in protein prediction, particularly the emergence of AlphaFold3—an AI model capable of predicting protein, RNA, and DNA structures—have demonstrated remarkable accuracy in modeling biomolecular interactions, including those involving small molecules [23, 24]. To investigate the binding mode and site of FMR1 with RNA, we performed structural comparisons and predictions for RNA fragments and full-length FMR1 using AlphaFold3.

**Fig. 1:**
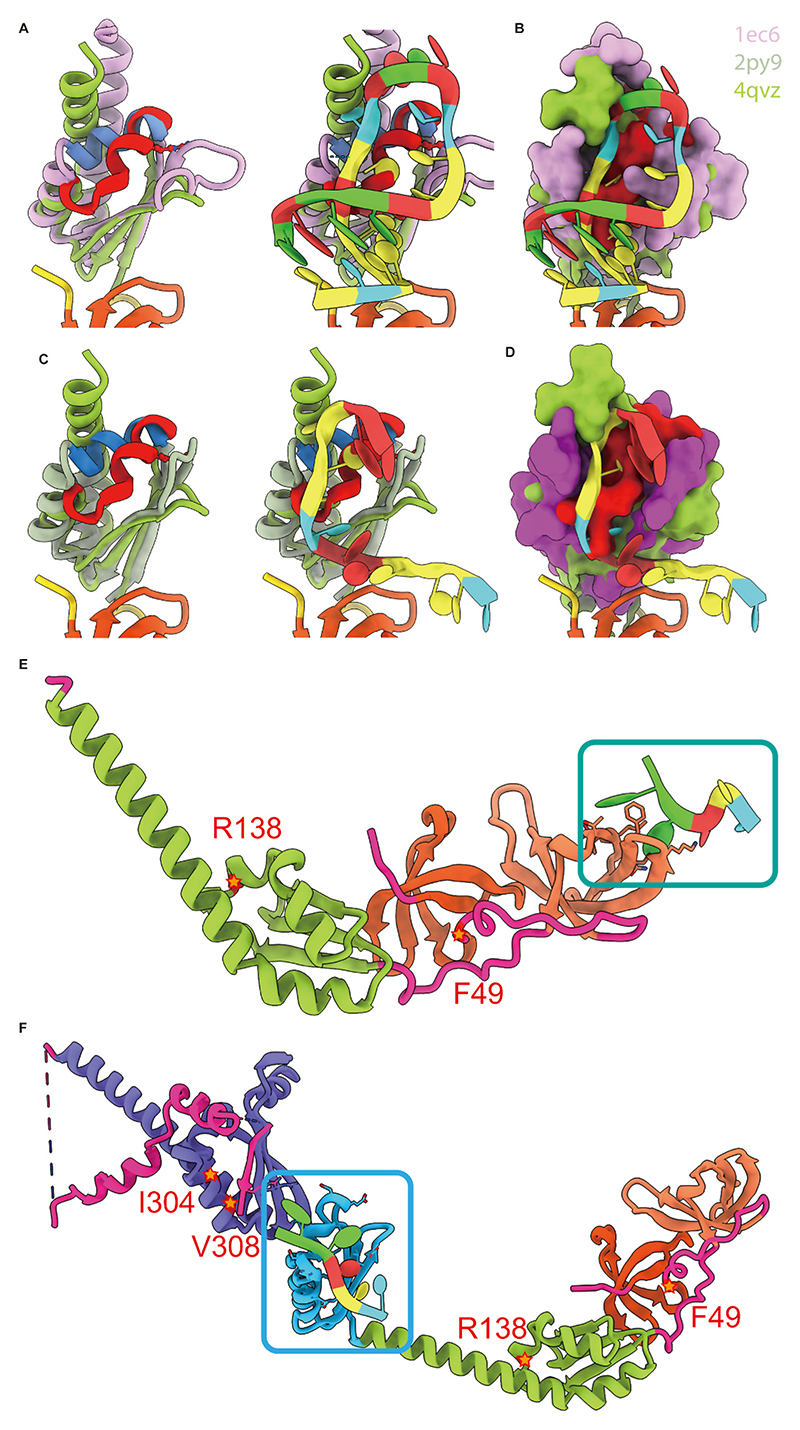
Structural comparison and AlphaFold3 predictions of FMR1 proteins. (A) Structural alignment of the KH domain in FMR1 (PDB: 4QVZ, green) and KH family protein 1EC6 (pink). Helices 1 and 2 of the KH0 domain (FMR1) are highlighted in red; corresponding regions in 1EC6 are blue. (B) Comparison of RNA-bound 4QVZ and 1EC6 structures, with the simulated solvent-accessible surface of the KH domain-RNA complex. Helices 1 and 2 in FMR1’s KH0 domain exhibit distinct RNA recognition modes versus 1EC6. (C) Structural alignment of FMR1 (4QVZ, green) and KH family protein 2PY9 (gray-green). KH0 domain helices 1 and 2 (red) are compared to corresponding regions in 2PY9 (blue). (D) Comparison of RNA-bound 4QVZ and 2PY9 structures, with the KH domain-RNA complex surface. FMR1’s KH0 domain helices 1 and 2 show divergent RNA recognition modes compared to 2PY9. (E) AlphaFold3-predicted complex of FMR1’s amino acid domain (residues 1–215) with RNA (GGACU). (F) AlphaFold3-predicted complex of full-length FMR1 with RNA (GGACU).

The KH domain, a conserved motif for nucleic acid recognition in cellular functional proteins, typically binds single-stranded RNA (ssRNA) and is integral to proteins regulating transcription, translation, and other cellular processes. We first compared the KH0 structure of FMR1 (PDB: 4QVZ) with the RNA-bound structures of two other KH family proteins (PDB: 1EC6, 2PY9) [8,10,25]. Although KH0 retains the canonical β-α-α-β-β-α folding of the KH family, helices 1 and 2 in KH0 are spatially offset relative to classical KH domains and positioned within the RNA-binding pocket of canonical KH proteins (Fig. 1A–D). This displacement likely restricts RNA access to KH0, suggesting it lacks direct RNA-binding capability.

To further explore FMR1-RNA interactions, we used AlphaFold3 to predict the complex structure between the N-terminal nucleic acid-binding region (residues 1–215) of FMR1 and the key RNA sequence GGACU. The model revealed a spatial arrangement resembling PDB: 4QWZ, with RNA binding at the terminus of the Age2 domain rather than the KH0 region (Fig. 1E).

Additionally, prediction of the full-length FMR1-RNA complex showed RNA binding localized to the KH1 domain (Fig. 1F). Collectively, these structural analyses and simulations indicate that the KH0 region is unlikely to directly engage in RNA binding.

### 2.2 The KH0 domain interacts with nucleic acids

To validate the predictive results from AlphaFold3, we performed biochemical experiments to characterize the interaction between FMR1 protein and RNA. Recombinant FMR1 protein and its mutant variants were generated via in vitro gene recombination. A pair of 42-nucleotide RNA probes—including both unmodified (ss-A) and m^6^A-modified (ss-m^6^A) versions—were designed, synthesized, and labeled with FAM and biotin for detection [26–28]. Using these probes, we conducted EMSA and SPR experiments to assess binding interactions. The following mutations were introduced to encompass key structural domains of FMR1 (Supplementary Fig. 1): FMR1^F49N^, FMR1^R138Q^, FMR1^I304N^, FMR1^V308K^, and FMR1^R534L&R546L^.

**Fig. 2:**
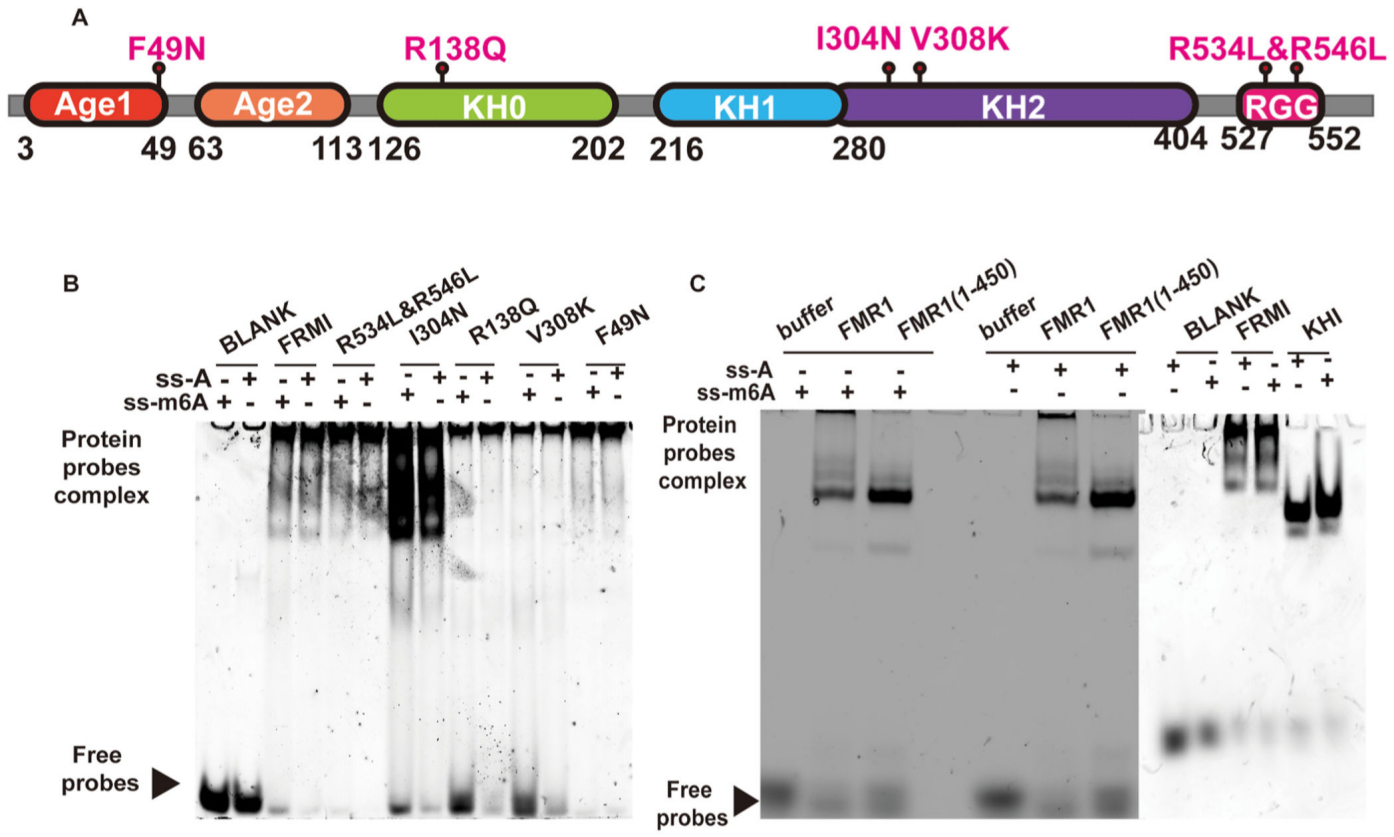
EMSA detection of wild-type/mutant FMR1 binding to m^6^A-modified/unmodified RNA. (A) Domain architecture of human FMR1: Age1 (red), Age2 (orange), KH0 (green), KH1 (blue), KH2 (purple), and RGG box (rose red). Mutation sites and domain boundaries are labeled. (B) EMSA analysis of wild-type/mutant FMR1 binding to RNA probes (with/without m^6^A modification). (C) EMSA analysis of full-length FMR1 or truncated variants (FMR1(1–450), FMR1(KH1)) binding to RNA probes (with/without m^6^A modification).

EMSA screening with the FMR1^WT^ protein and these mutants revealed distinct binding patterns (Fig. 2A). FMR1^WT^ exhibited robust interaction with both ss-A and ss-m^6^A probes. Similarly, mutants FMR1^R534L&R546L^ and FMR1^F49N^ maintained strong binding to both probe types. In contrast, mutations at FMR1^R138Q^, FMR1^I304N^, and FMR1^V308K^ significantly impaired binding to ss-m^6^A probes but did not affect binding to ss-A probes (Fig. 2B).

To further map RNA-binding regions, we analyzed FMR1(1–450) and FMR1(KH1) (Fig. 2C) truncation mutants via EMSA. These results demonstrated that RNA-binding sites are distributed across the entire FMR1 protein. Notably, FMR1^R138Q^, FMR1^I304N^, and FMR1^V308K^ were identified as novel key binding sites specifically mediating interactions with ss-m^6^A, suggesting potential synergistic effects between these sites and other regions of FMR1 during nucleic acid binding.

**Fig. 3:**
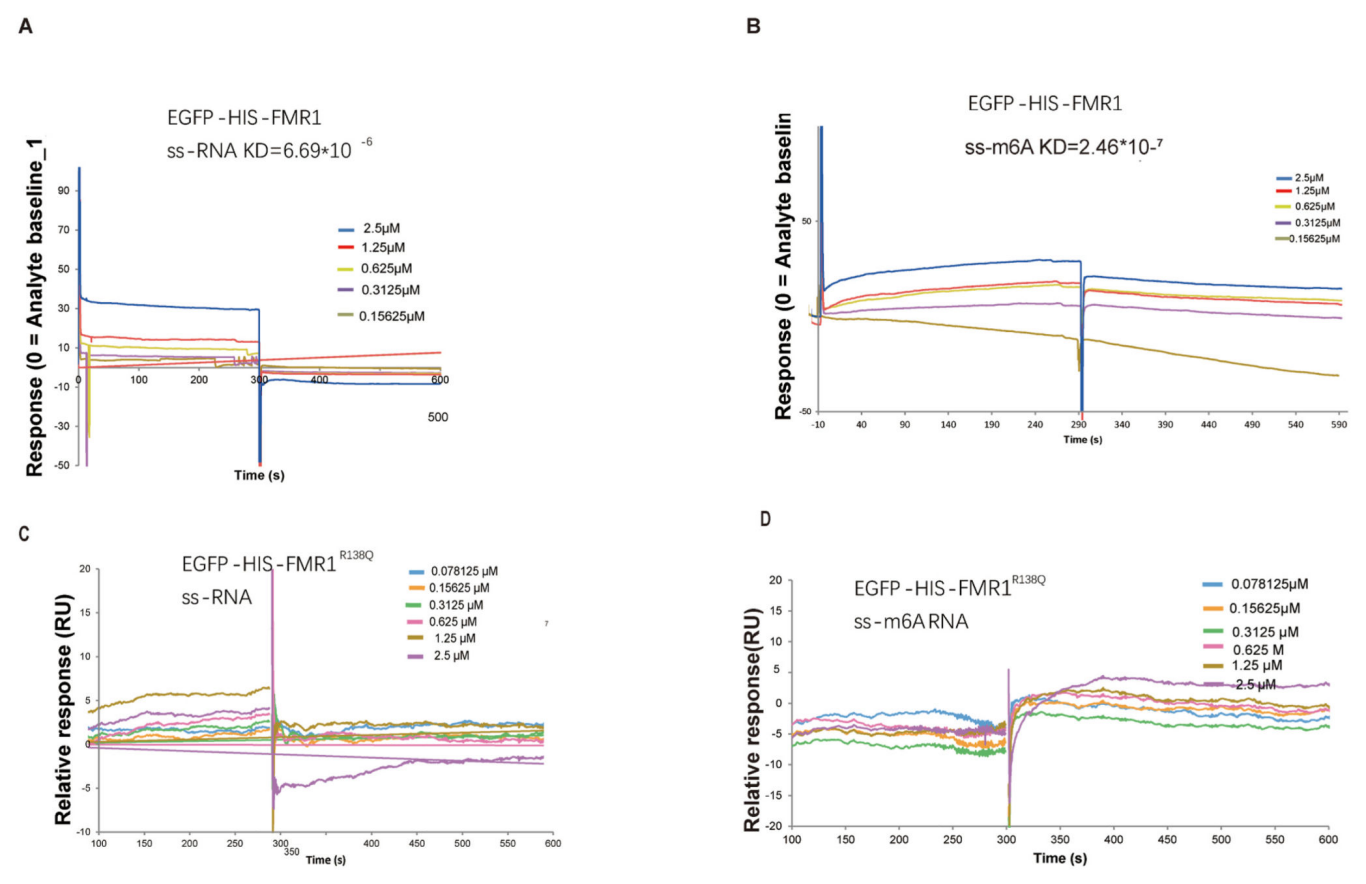
SPR detection of wild-type/mutant FMR1 binding to m^6^A-modified/unmodified RNA. (A, B) SPR binding curves for FMR1^WT^ protein with unmodified (A) or m^6^A-modified (B) RNA probes. (C, D) SPR binding curves for FMR1^R138Q^ protein with unmodified (C) or m^6^A-modified (D) RNA probes. Equilibrium (KD) and kinetic constants were calculated via global fitting to the 1:1 Langmuir model. Lower KD values indicate higher binding affinity.

**Fig. 4:**
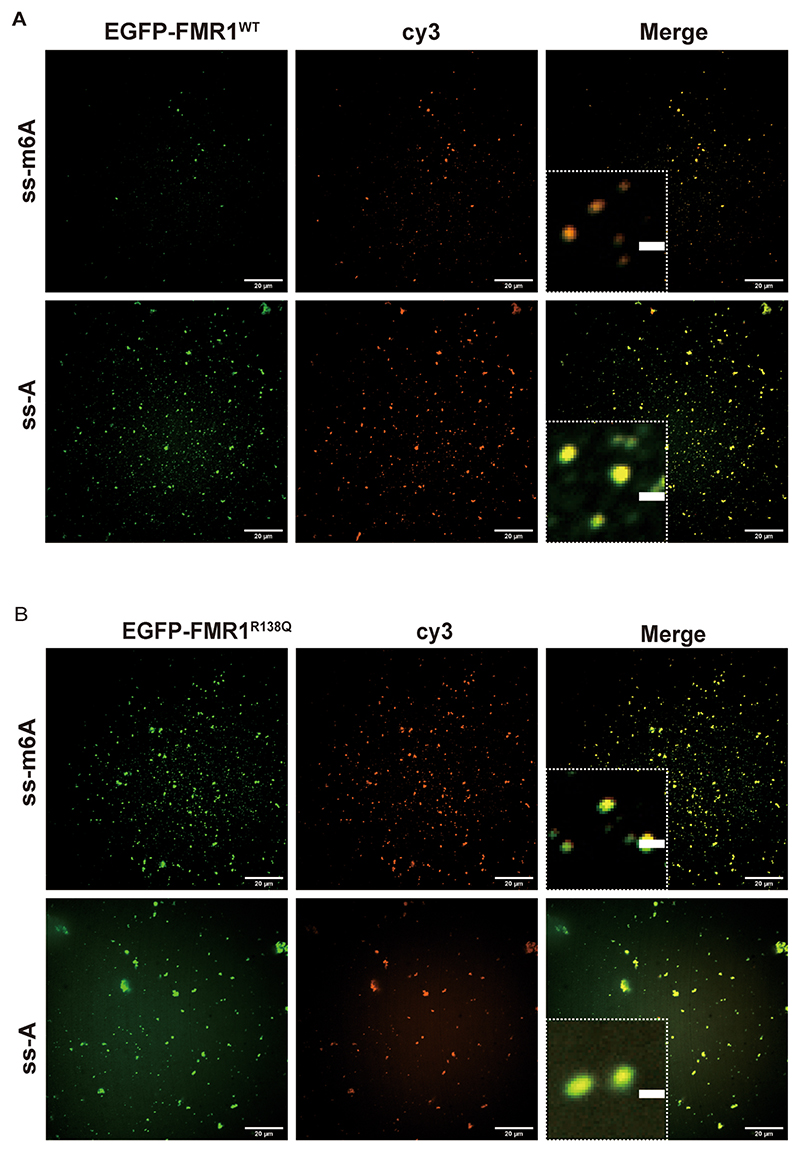
Co-localization of FMR1^WT^/mutant FMR1^R138Q^ with m^6^A-modified/unmodified RNA in phase-separated droplets. (A, B) Both FMR1^WT^ and FMR1^R138Q^ form droplets containing m^6^A-modified or unmodified RNA in vitro.

SPR experiments further confirmed these findings. Binding assays between FMR1^WT^ and the R138 site in the KH0 region showed that FMR1^WT^ exhibited stronger binding affinity for ss-m^6^A than for ss-A (Fig. 3A and B). Importantly, the R138Q mutation significantly disrupted the interaction between FMR1 and ss-m^6^A probes (Fig. 3C and D). Microscopic co-localization studies revealed that both wild-type FMR1 and FMR1^R138Q^ mutants co-localized with ss-A probes, regardless of mutation status (Fig. 4A and B).

Collectively, these data indicate that the KH0 region of FMR1 interacts with both ss-A and ss-m^6^A, potentially through direct binding sites or cooperative interactions with other domains. FMR1 exhibits preferential binding to m^6^A-modified RNA, with the KH2 region harboring established binding sites for m^6^A-RNA. Mutations (FMR1^R138Q^, FMR1^I304N^, FMR1^V308K^) synergistically inhibit m^6^A-RNA binding without disrupting protein-RNA co-localization.

Comparative analysis of AlphaFold3 predictions and experimental results revealed discrepancies. AlphaFold3 predicted that the primary RNA-binding sites of FMR1 would localize to the Age2 and KH1 regions; however, mutations in these regions did not significantly alter RNA-binding capacity in vitro. In contrast, mutations in FMR1^R138Q^, FMR1^I304N^, and FMR1^V308K^ reduced binding affinity for methylated RNA. This suggests that m^6^A modification may influence the KH0 and KH2 regions, while the KH1 region remains largely unaffected.

Since AlphaFold3 cannot simulate m^6^A-RNA-protein interactions, we propose that the reduced binding affinity of methylated RNA to mutants (FMR1^R138Q^, FMR1^I304N^, FMR1^V308K^) likely stems from two factors: (1) Direct disruption of binding sites by mutations, and (2) Altered inter-domain cooperativity that facilitates nucleic acid binding. Methylation may modify the cooperative interactions between domains, leading to differential binding outcomes for methylated versus unmodified RNA. Specifically, mutations FMR1^I304N^ and FMR1^V308K^ in the KH2 region may alter surface charge distribution, disrupting the charge balance within the hydrophobic network and thereby modulating nucleic acid binding affinity.

### 2.3 Phase separation of FMR1

Aberrant phase separation in biological systems is increasingly recognized as a driver of disease pathogenesis. Within phase-separated droplets, protein condensation can yield pathological solid-like aggregates, implicating phase separation dysregulation in disease mechanisms. Targeting phase separation—through interference, modulation, or disruption—thus emerges as a promising therapeutic strategy. Beyond its pathological implications, protein phase separation plays critical roles in cellular organization, tissue integrity, and signal transduction [29–31].

As a core component of dynamically transported neuronal granules (shuttling from somatic cells to dendrites), FMR1 belongs to the RNA-binding protein family [32–34]. Its structural features—hydrophobic networks, π-π interaction interfaces, and high arginine content—strongly predict phase separation propensity [35].

**Fig. 5:**
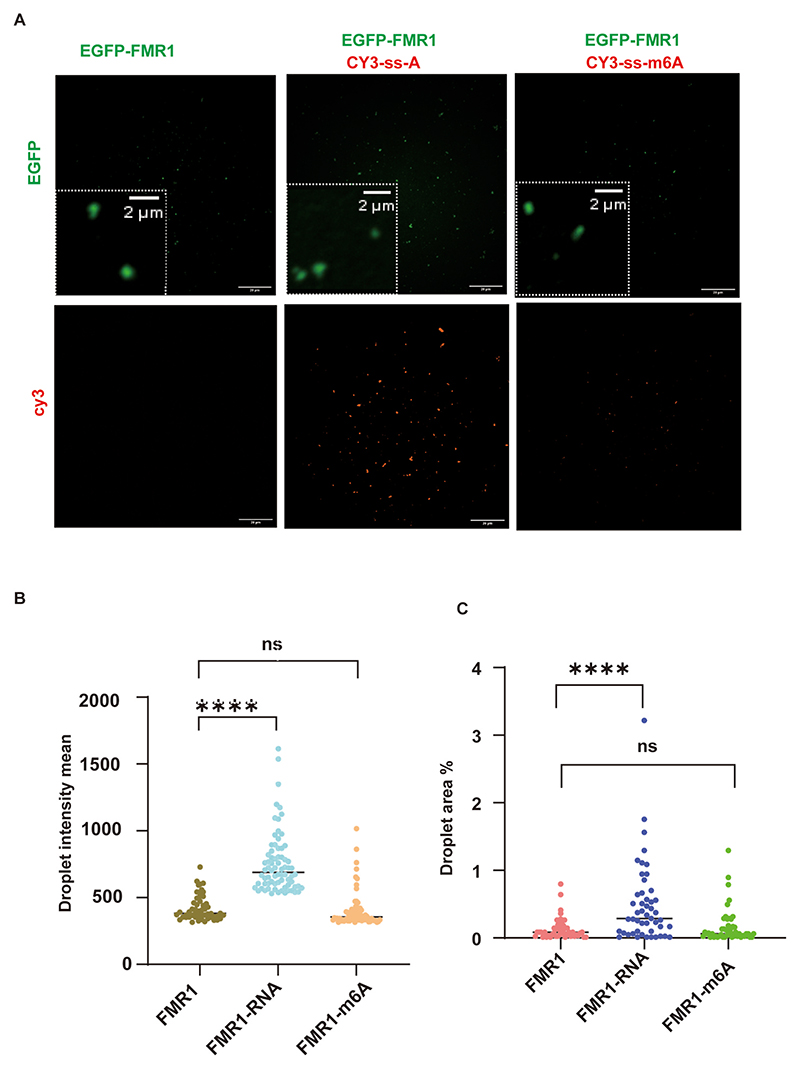
RNA-dependent droplet formation by FMR1^WT^ in vitro. (A) Droplet formation analysis of GFP-FMR1^WT^ mixed with unmodified RNA (Cy3-ss-A) or m^6^A-modified RNA (Cy3-ss-m^6^A). Scale bar: 5 μm. (B) Quantified green droplet area (GFP-FMR1^WT^+Cy3-ss-A: ***p < 0.001; GFP-FMR1^WT^+Cy3-ss-m^6^A: P = 0.47). (C) Quantified green droplet fluorescence intensity (GFP-FMR1^WT^+Cy3-ss-A: ****p < 0.0001; GFP-FMR1^WT^+Cy3-ss-m^6^A: *p* = 0.37).

To investigate FMR1’s phase separation behavior, we examined changes in protein particle size and polymerization dynamics upon nucleic acid co-localization in vitro. Confocal microscopy revealed that FMR1^WT^ forms condensates at critical concentrations in solution. Addition of ss-A RNA significantly increased the size of FMR1^WT^ droplets, whereas ss-m^6^A exhibited negligible effects on droplet size (Fig. 5).

**Fig. 6:**
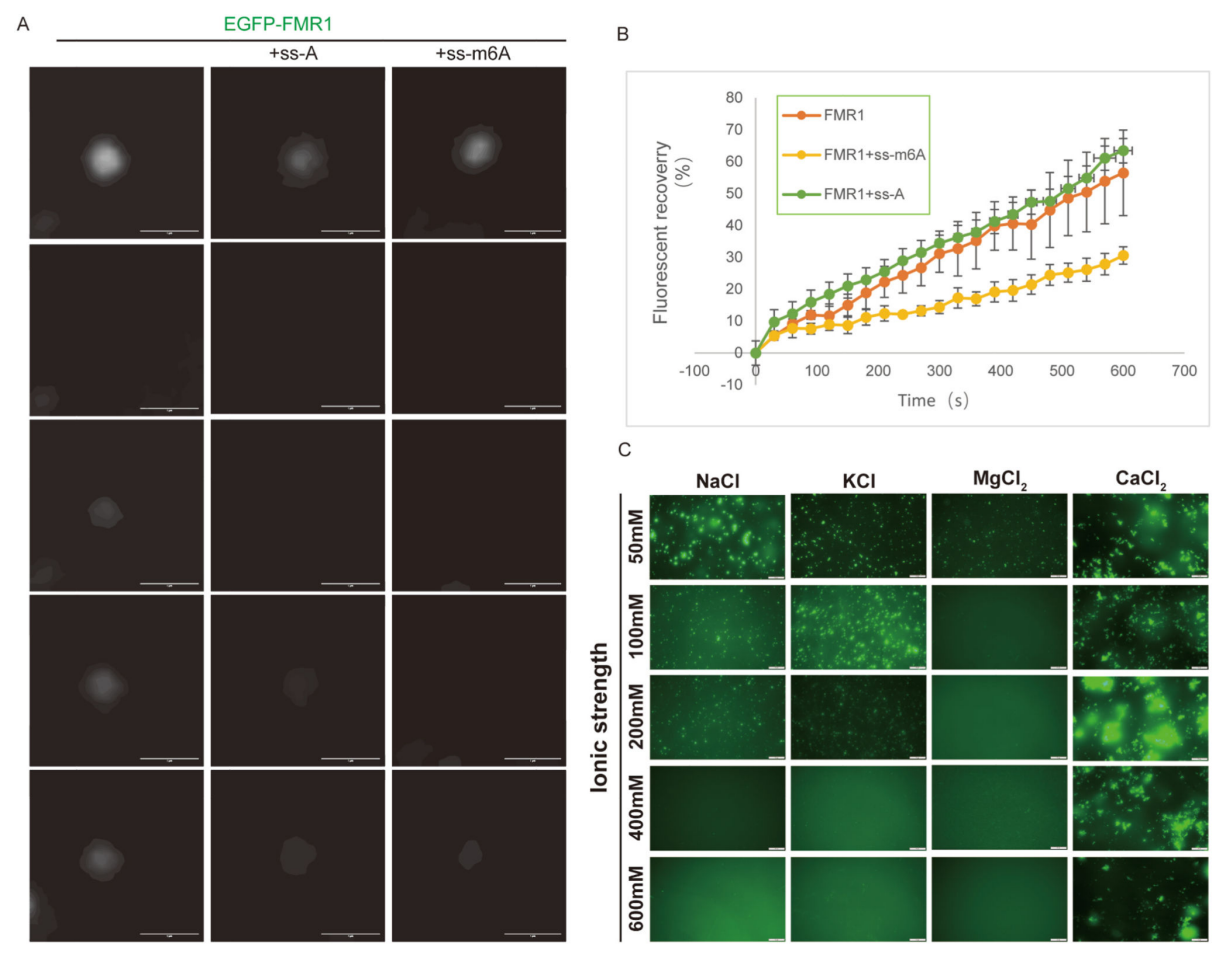
m^6^A modification and salt effects on FMR1^WT^ droplet fluidity. (A) FRAP analysis of FMR1^WT^ droplet fluidity. Three independent images show droplet recovery at different time points post-photobleaching. Scale bar: 1 μm. (B) Fluorescence recovery curves for droplets with unmodified RNA or m^6^A-RNA (mean ± SD, n = 3). (C) FMR1^WT^ droplet images under varying salt concentrations (10 μM protein, 25 μM Na_2_HPO_4_, pH 7.4, 2 mM DTT).

Fluorescence recovery after photobleaching (FRAP) further elucidated the impact of RNA binding on condensate fluidity. Compared to droplets formed by FMR1^WT^ alone or with ss-A, FRAP analysis demonstrated that FMR1^WT^-ss-m^6^A droplets exhibited the lowest recovery rate (Fig. 6A and B), indicating that ss-m^6^A binding markedly reduces the fluidity of FMR1^WT^ condensates.

We previously established that FMR1 phase separation occurs independently of additives. To disrupt electrostatic interactions within FMR1^WT^ droplets, we mapped phase diagrams across varying protein and salt concentrations via microscopy. Increased salt concentrations were found to suppress FMR1^WT^ droplet formation, a phenomenon persisting across mutant proteins, diverse salt ions, and nucleic acid conditions (Fig. 6C, Supplementary Fig. 2). These findings highlight the critical role of ionic strength in regulating droplet formation and maintaining protein polymerization states—a prerequisite for structural analysis.

Collectively, our data demonstrate that FMR1 forms phase-separated condensates in a polymeric state. Notably, m^6^A-modified RNA inhibits the assembly of high-order protein complexes, thereby facilitating normal translation. This study provides direct evidence for FMR1 phase separation and reveals that ss-m^6^A binding selectively impairs protein re-aggregation, offering mechanistic insights into RNA-mediated regulation of FMR1 function.

### 2.4 FMR1^R138Q^ reduces the interaction with ss-m^6^A

Pathological mutations in the FMR1 protein disrupt its physiological functions, contributing to neurodevelopmental disorders. A missense mutation substituting arginine 138 with glutamine (R138Q) has been identified in patients with intellectual disability and epileptic seizures [36,37]. This residue resides within the KH0 domain—a region under-explored for its structural and functional properties. Investigating FMR1^R138Q^, a variant linked to disease phenotypes, is critical for understanding m^6^A-mediated regulation of FMR1 phase separation.

**Fig. 7:**
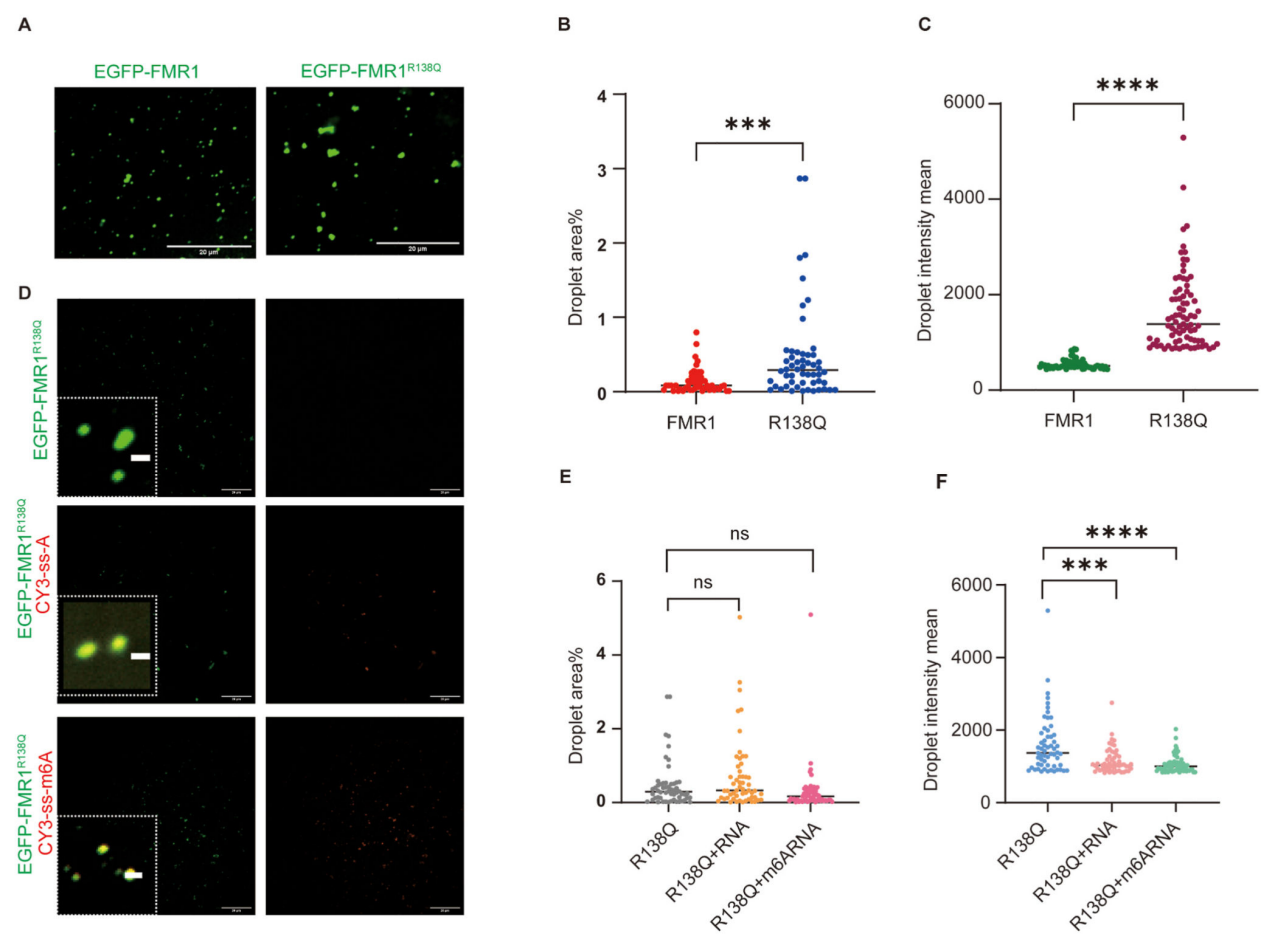
Impact of FMR1^R138Q^ on droplet size and m^6^A-dependent changes. (A) Comparison of droplet size for FMR1^R138Q^ and GFP-FMR1^WT^ (without RNA or with unmodified/m^6^A RNA). Scale bar: 5 μm. Quantified area/intensity: (B) Area (***p < 0.0002); (C) Intensity (****p < 0.0001). (D) Droplet formation assay of GFP-FMR1^R138Q^ with unmodified RNA (Cy3-ss-A) or m^6^A-RNA (Cy3-ss-m^6^A). Scale bar: 5 μm. (E) Quantified green droplet area (GFP-FMR1^R138Q^+Cy3-ss-A: *p* = 0.13; GFP-FMR1^R138Q^+Cy3-ss-m^6^A: *p* = 0.17). (F) Quantified green droplet intensity (GFP-FMR1^R138Q^+Cy3-ss-A: ***p < 0.0002; GFP-FMR1^R138Q^+Cy3-ss-m^6^A: ****p < 0.0001).

Confocal microscopy revealed that FMR1^R138Q^ and wild-type FMR1 (FMR1^WT^) form condensates at identical concentrations in 50 mM NaCl. Notably, FMR1^R138Q^ exhibited enhanced phase separation capacity, manifesting as larger droplet size and elevated fluorescence intensity compared to FMR1^WT^ (Fig. 7A–C). Addition of ss-A or ss-m^6^A did not significantly alter droplet size. However, ss-m^6^A markedly increased the fluorescence intensity of FMR1^R138Q^ condensates, while ss-A had negligible effects (Fig. 7D–F).

**Fig. 8:**
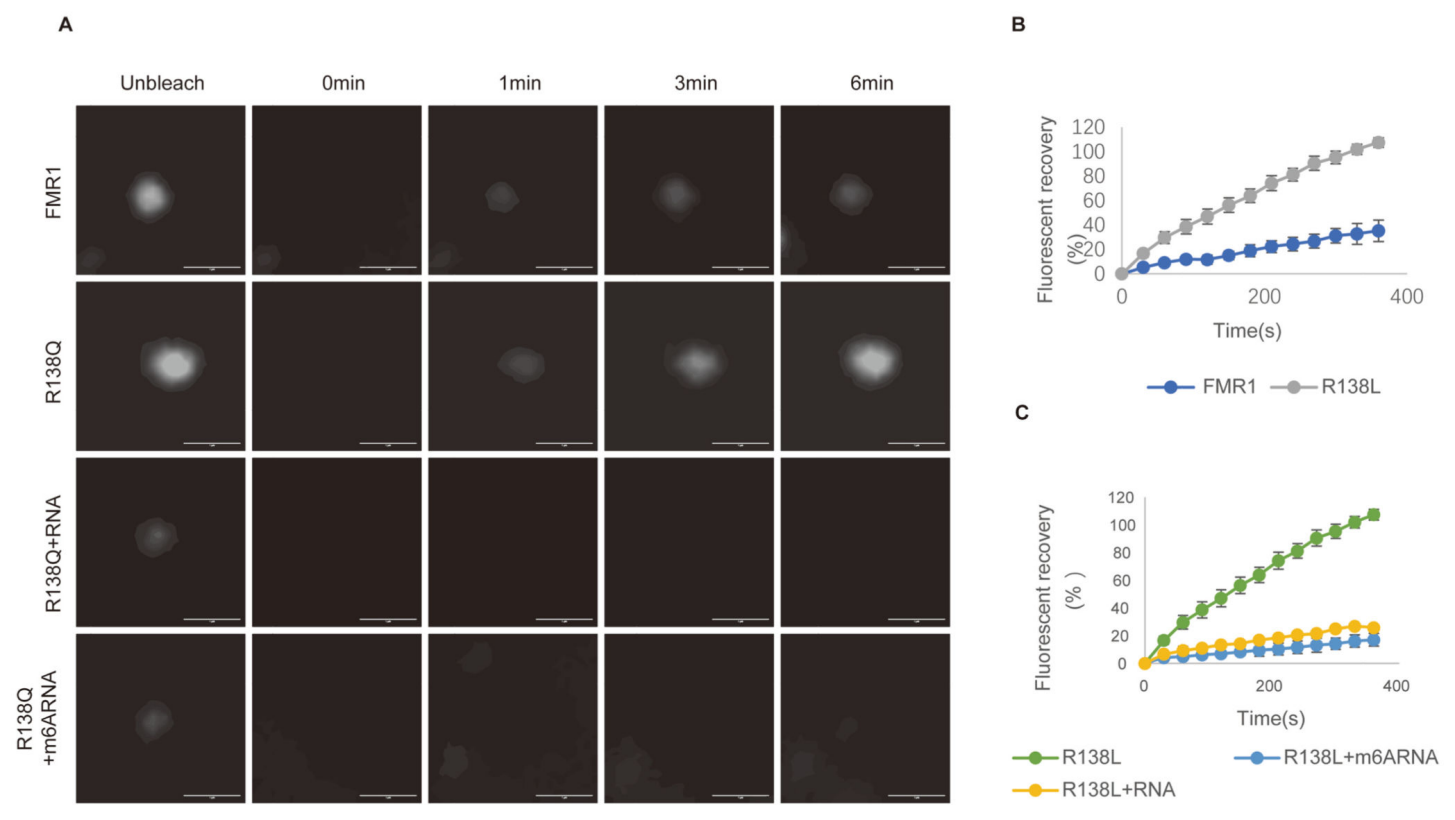
m^6^A-dependent fluidity of FMR1^R138Q^ droplets. (A) FRAP analysis of GFP-FMR1^WT^ (no RNA), GFP-FMR1^R138Q^ (no RNA), and GFP-FMR1^R138Q^ (with unmodified/m^6^A RNA). Time-course images show droplet recovery. Scale bars: 1 μm. (B) Fluorescence recovery curves for FMR1^WT^ and FMR1^R138Q^ droplets (mean ± SD, n = 3). (C) Fluorescence recovery curves for FMR1^R138Q^ droplets with unmodified RNA or m^6^A-RNA (mean ± SD, n = 3).

FRAP analysis further demonstrated that the R138Q mutation significantly enhanced droplet fluidity under basal conditions (Fig. 8). Strikingly, addition of ss-A or ss-m^6^A rapidly reduced the fluidity of FMR1^R138Q^ droplets, with ss-m^6^A exhibiting a more pronounced effect than ss-A.

Collectively, these data indicate that FMR1^R138Q^ exhibits reduced binding affinity for ss-m^6^A, leading to aberrant phase separation characterized by excessive droplet aggregation. While ss-A and ss-m^6^A minimally impact droplet size, they differentially modulate condensate fluidity: ss-m^6^A accelerates droplet disassembly, whereas ss-A has negligible effects. This suggests that m^6^A modification selectively regulates FMR1^R138Q^ phase behavior, potentially through disrupted protein-RNA interactions.

## 3 Discussion

### 3.1 Conservation of FMR1 function and disease relevance

Our study demonstrates that human FMR1 shares functional conservation with its Drosophila counterpart, particularly in recognizing m^6^A-modified “AGACU” motifs to regulate RNA dynamics [38]. This conservation extends to disease mechanisms, as mutations in FMR1—such as R138Q in the KH0 domain—are linked to Fragile X Syndrome (FXS). The R138Q mutation impairs m^6^A-RNA binding, increases droplet size, and reduces fluidity, leading to aberrant phase separation and protein aggregation. These findings underscore the critical role of methylated RNA recognition in FMR1’s physiological function and highlight its dysfunction as a key driver of FXS pathogenesis.

### 3.2 Discrepancies between predictions and experimental results

Unexpectedly, our biochemical data revealed that the KH0 domain—predicted by AlphaFold3 to lack nucleic acid-binding capability—plays a direct role in m^6^A-RNA interactions. While AlphaFold3 localized binding to the KH1 region, mutations in R138 (within KH0) significantly altered m^6^A-RNA binding affinity. This discrepancy suggests that FMR1-RNA interactions may involve cooperative effects between multiple domains or dynamic conformational changes induced by methylation, which current AI models cannot fully capture.

### 3.3 Phase separation as a therapeutic target

Our phase separation analyses demonstrate that m^6^A-RNA binding modulates the fluidity and size of FMR1 droplets, while salt concentration changes disrupt droplet formation. These findings highlight phase separation as a potential therapeutic target for FXS. Controlling droplet dynamics through pharmacological or biophysical interventions could restore normal protein function and mitigate disease phenotypes.

### 3.4 Limitations and future directions

Despite these advances, several challenges remain. AlphaFold3’s inability to predict modified RNA-protein interactions limits its utility in studying epitranscriptomic regulation. Additionally, the structural basis for KH0’s unexpected role in m^6^A binding remains unclear, as its secondary structure is preserved despite mutation.

Future research should focus on.

- Cryo-EM structural studies to resolve full-length FMR1-nucleic acid complexes and identify methylation-sensitive binding sites.
- Functional validation in Drosophila using humanized FMR1 mutants to bridge molecular findings with behavioral phenotypes.
- Therapeutic development targeting phase separation or RNA-binding affinity to restore normal FMR1 function in FXS.

## 4 Conclusion

By integrating computational predictions, biochemical assays, and biophysical analyses (Supplementary Figs. 3 and 4), our study reveals the complex interplay between FMR1, m^6^A-RNA, and phase separation. The unexpected contribution of KH0 to methylated RNA binding highlights the limitations of current AI tools and the need for multidisciplinary approaches. These insights provide a foundation for understanding FXS pathogenesis and developing targeted therapies to address RNA-binding protein dysregulation in neurodevelopmental disorders.

## 5 Materials and methods

### 5.1 Protein structure prediction and analysis

Three previously published protein structures (PDB IDs: 1EC6, 2PY9, 4QVZ) were used for structural comparison. Alignments and visualizations were performed in ChimeraX. RNA-binding models of FMR1 with the sequence GGACU were predicted using AlphaFold3. For each prediction set, Model 0 was selected for further analysis.

### 5.2 Expression and purification of FMR1 and mutant proteins

The full-length human FMR1 gene was cloned into a vector with an N-terminal 6 × His-tag and EGFP tag under the control of a lac1 promoter. Protein expression was performed in Transetta (DE3) cells induced with 1 mM IPTG at 14 °C for 18 h. Harvested cells were lysed by centrifugation (4000 rpm, 15 min), and the supernatant was incubated with Ni-agarose resin (Qiagen). After washing with lysis buffer (500 mM NaCl, 50 mM Tris-HCl [pH 8.0], 10 % glycerol, 40 mM imidazole), FMR1 was eluted with 250 mM imidazole and 1 mM β-mercaptoethanol (β-Me). The protein was concentrated to ~2 mg/mL in storage buffer (250 mM NaCl, 25 mM Tris-HCl [pH 8.0]).

### 5.3 Electrophoretic mobility shift assays (EMSAs)

FAM-labeled RNA probes (FAM-AUGGGCCGUUCAU-CUGCUAAAAGGXCUGCUUUUGGGGCUUGU-3’, X = A or m^6^A) were synthesized by Tsingke Biotechnology. FMR1 protein was incubated with RNA probes in reaction buffer (25 mM Tris-HCl [pH 8.0], 150 mM NaCl, 50 mM KCl, 5 mM MgCl_2_, 10 mM DTT, 1 μM BSA) for 30 min at room temperature. Complexes were resolved on an 8 % native polyacrylamide gel (0.5 × TBE buffer, 120 V, 35 min) and visualized using a Typhoon FLA9500 scanner (GE Healthcare).

### 5.4 Surface plasmon resonance (SPR) measurements

Biotinylated RNA probes (biotin-UGGGCCGUUCAU-CUGCUAAAAGGXCUGCUUUUGGGGCUUGU-3’, X = A or m^6^A) were immobilized on SA chips (Cytiva). Binding assays were performed on a Biacore 8K instrument (GE Healthcare) at 25 °C. FMR1 protein was injected at 30 μL/min (120 s association, 300 s dissociation) in running buffer (1 × PBS, 0.1 % BSA, 0.02 % Tween 20). Binding constants were calculated using the 1:1 Langmuir model in Biacore 8K Evaluation Software.

### 5.5 Phase separation assay

GFP-tagged FMR1^WT^ and FMR1^R138Q^ proteins were expressed in *E. coli* and purified. Phase separation was induced by mixing 10 μM protein with buffer (20 mM Tris-HCl [pH 8.0], 50 mM NaCl, 25 mM Na_2_HPO_4_). For RNA-dependent assays, 5 μM Cy3-labeled A/m^6^A RNA was added. Droplets were imaged on a Nikon CSU SORA confocal microscope (60 × oil lens, 488 nm/405 nm lasers). DIC images were acquired using an OLYMPUS ix73 microscope (100 × oil lens).

### 5.6 Fluorescence recovery after photobleaching (FRAP)

Droplet fluidity was assessed by photobleaching GFP-labeled droplets (488 nm laser, 50 % intensity) on a Nikon CSU SORA confocal microscope. Images were captured every 30 s for 6 min. Recovery ratios were calculated from ≥5 spots across three independent experiments by comparing bleached and unbleached regions.

## Supplementary Material

Supplementary data to this article can be found online at https://doi.org/10.1016/j.yexcr.2025.114664.

Supplementary file

## Data Availability

Data will be made available on request.
