# Supplementary material for: FMR1 KH0-KH1 domains coordinate m6A binding and phase separation in Fragile X syndrome
Source: Exp Cell Res. Author manuscript; Available in PMC 2026 Apr 14. (PMC7619003; doi:10.1016/j.yexcr.2025.114664)
Supplement: Supplementary file [file EMS213044-supplement-Supplementary_file.pdf]

FMR1 KH0-KH1 Domains Coordinate m6A Binding and Phase Separation in Fragile X Syndrome

Xian Zhou[1,2], Chen-Jun Guo[2], Rui Wang [2], Yi-Lan Li[2], Tianyi Zhang[2], Zhuangyi Qiu[2], Shaorong Gao[1,3,*], Ji-Long Liu[2,4*], Yawei Gao[1,5, *]

Supplementary Information

Supplimentart figures S1-S4 and figure legends

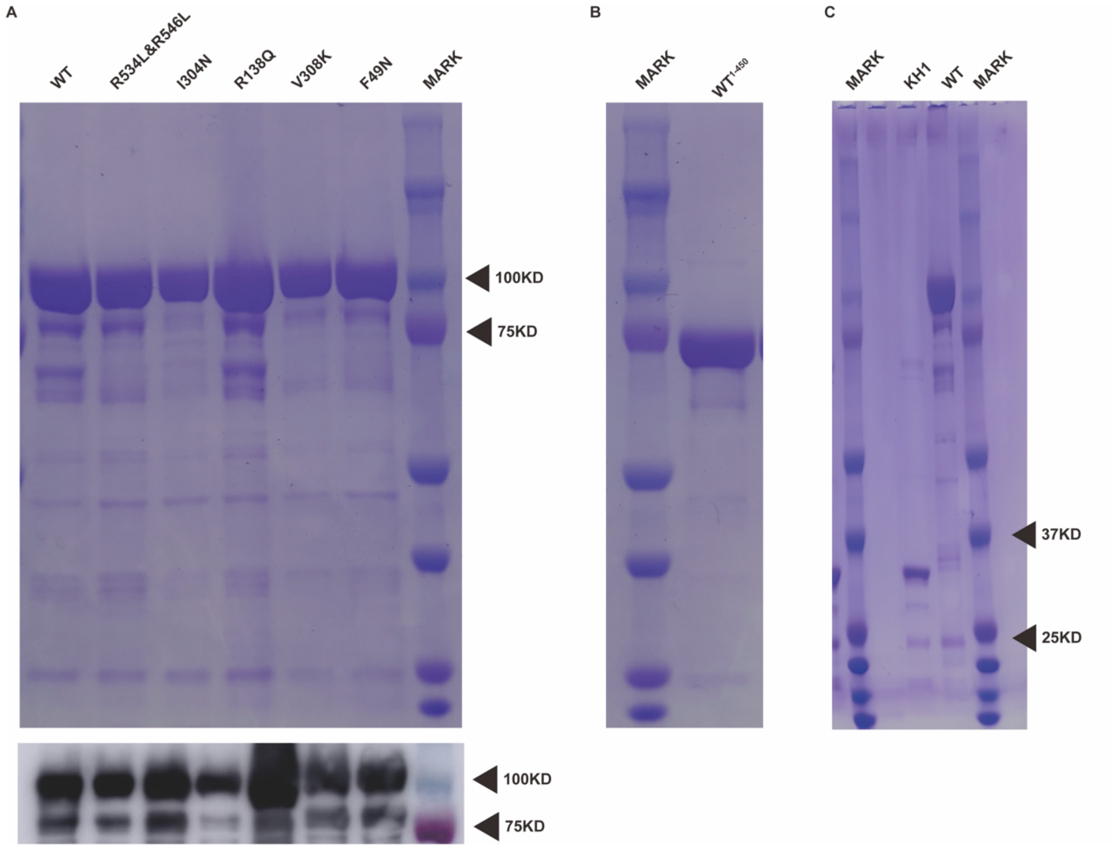

Supplementary Figure 1. Purification of human FMR1 protein and its mutants. (A) SDS-PAGE analysis of His-EGFP-FMR1 and mutant proteins stained with Coomassie Blue, alongside corresponding Western Blot results. (B) SDS-PAGE analysis of His-EGFP-FMR1 (residues 1–450) stained with Coomassie Blue. (C) SDS-PAGE analysis of His-EGFP-FMR1 KH1 domain stained with Coomassie Blue.

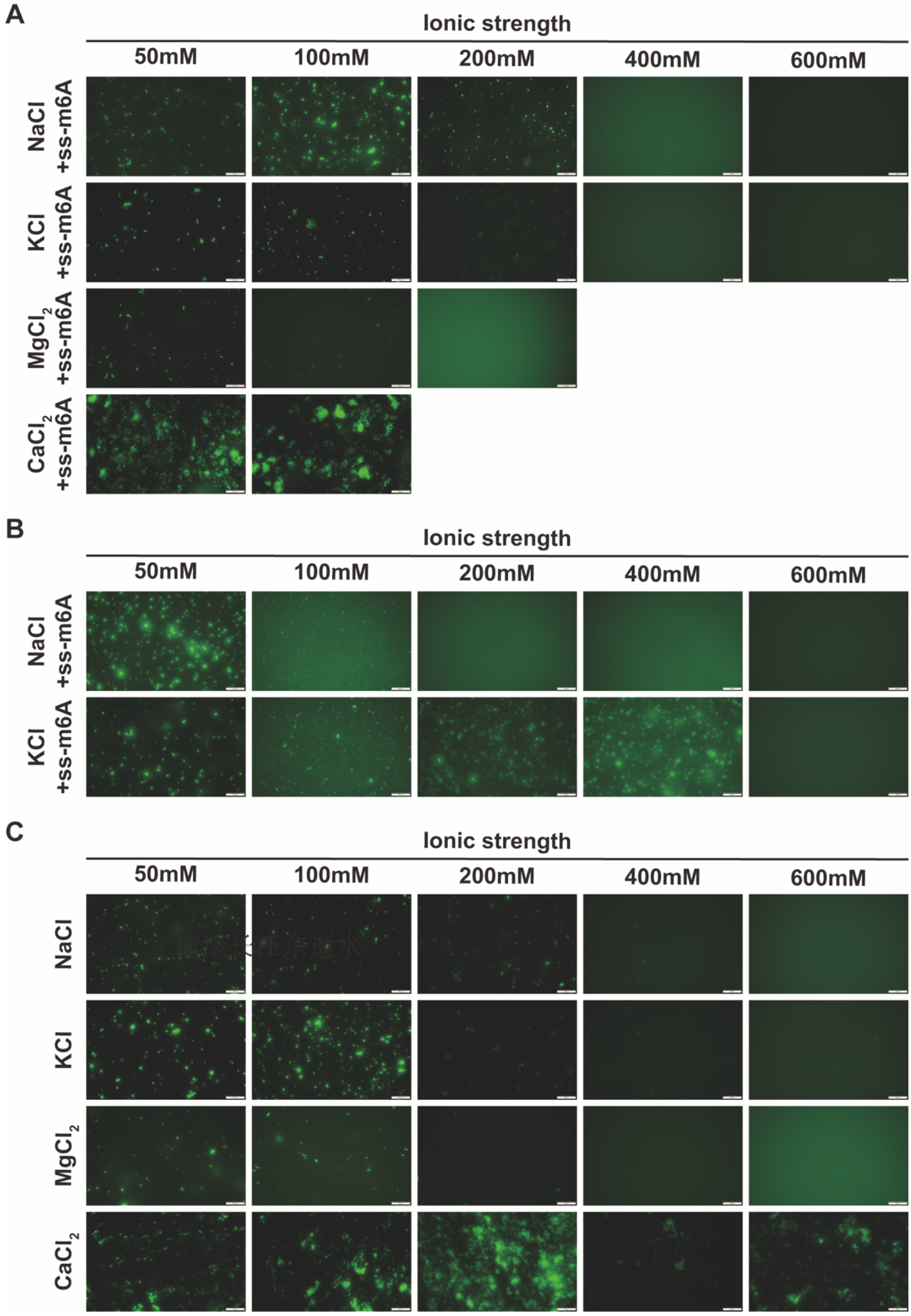
A
Ionic strength
50mM
100mM
200mM
400mM
600mM
NaCl +ss-m6A
KCl +ss-m6A
MgCl2 +ss-m6A
CaCl2 +ss-m6A
B
Ionic strength
50mM
100mM
200mM
400mM
600mM
NaCl +ss-m6A
KCl +ss-m6A
C
Ionic strength
50mM
100mM
200mM
400mM
600mM
NaCl
KCl
MgCl2
CaCl2

Supplementary Figure 2. Effect of salt ions on FMR1^{WT} and FMR1^{R138Q} droplet formation. (A) Droplet formation images of FMR1^{WT} (10 μM protein, 1 μM m^{6}A-RNA, 25 μM Na_2PO_4 pH 7.4, 2 mM DTT) under varying ion concentrations. (B) Droplet formation images of FMR1^{R138Q} (10 μM protein, 1 μM m^{6}A-RNA, 25 μM Na_2PO_4 pH 7.4, 2 mM DTT) with KCl and NaCl at different concentrations. (C) Droplet formation images of FMR1^{R138Q} (10 μM protein, 25 μM Na_2PO_4 pH 7.4, 2 mM DTT) under varying ion concentrations (without RNA).

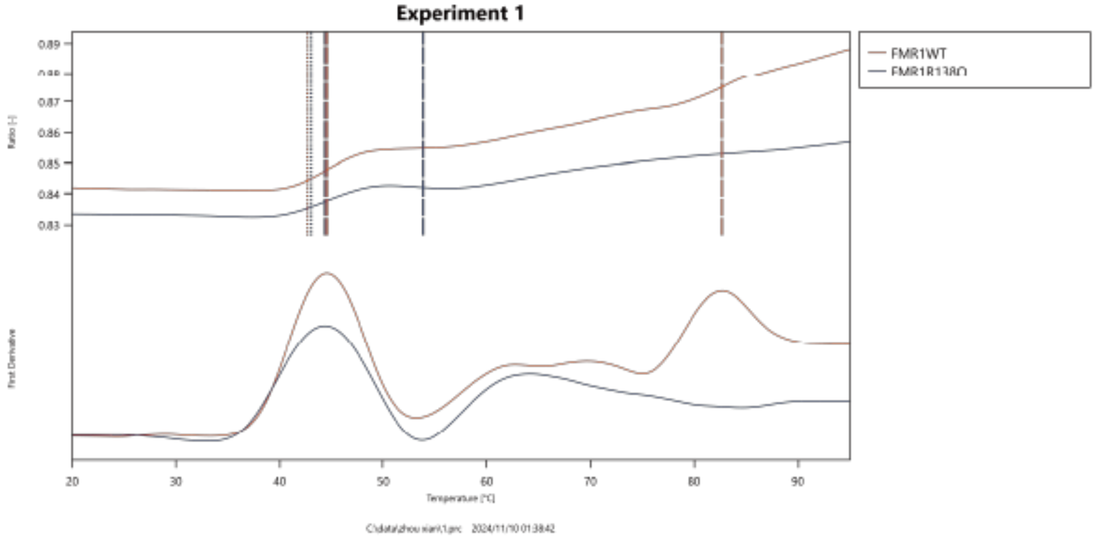

Sample ID	Onset #1 for Ratio	Inflection Point #1 for Ratio
FMR1WT	42.7°C	44.6°C
FMR1R138Q	43.0°C	44.4°C

Supplementary Figure 3. Melting temperature (Tm) analysis of FMR1^{WT} and FMR1^{R138Q} by Prometheus NT.48 nanoDSF. Thermodynamic parameters were measured using the Prometheus NT.48 nanoDSF system. "Onset#1 for Ratio" values represent Tm.

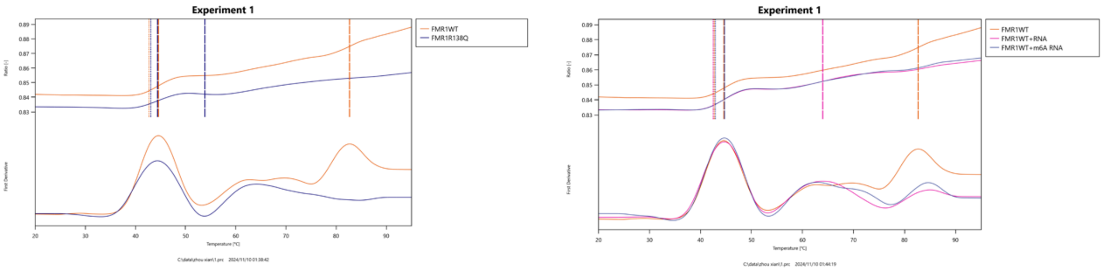

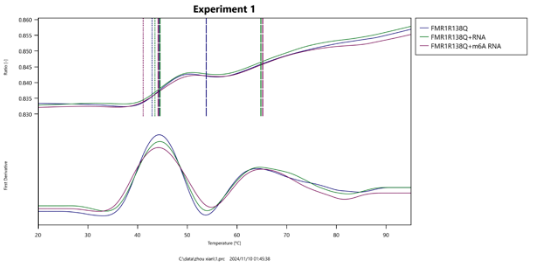

Sample ID	Onset #1 for Ratio	Inflection Point #1 for Ratio
FMR1WT	42.7°C	44.6°C
FMR1WT+RNA	42.5°C	44.6°C
FMR1WT+m6A RNA	42.9°C	44.7°C
FMR1R138Q	43.0°C	44.4°C
FMR1R138Q+RNA	43.5°C	44.5°C
FMR1R138Q+m6A RNA	41.2°C	44.2°C

Supplementary Figure 4. Melting temperature (Tm) analysis of FMR1^{WT} and FMR1^{R138Q} in complex with RNA by Prometheus NT.48 nanoDSF. Thermodynamic parameters were measured using the Prometheus NT.48 nanoDSF system. "Onset#1 for Ratio" values represent Tm.
